# Implementing a Metabolism-Informed Approach for Smoking Cessation in an Alaska Tribal Health System: Study Protocol for a Single-Arm Implementation Pilot Trial

**DOI:** 10.21203/rs.3.rs-3874126/v1

**Published:** 2024-01-23

**Authors:** Kelley Jansen, Brianna Tranby, Aliassa Shane, Todd Takeno, Kelly Chadwick, Pamela Sinicrope, Jennifer Shaw, Rachel Tyndale, Jeffrey Harris, Christi Patten, Jaedon Avey

**Affiliations:** Southcentral Foundation; Mayo Clinic; Southcentral Foundation; Southcentral Foundation; University of Washington; Mayo Clinic; University of Alaska Fairbanks; University of Toronto; University of Washington; Mayo Clinic; Southcentral Foundation

**Keywords:** Alaska Native and American Indian, Health Services, Tobacco Cessation, Implementation, Nicotine Metabolite Ratio

## Abstract

**Background:**

Individualized treatment for commercial tobacco smoking cessation, such as through the utilization of the nicotine metabolite ratio (NMR), offers substantial clinical benefit. NMR is a metabolism-informed biomarker that can be used to guide medication selection. NMR testing is particularly promising for tobacco cessation efforts in populations with high rates of smoking, such as some Alaska Native and American Indian (AN/AI) communities. To date, no prior study has evaluated the implementation of NMR-guided tobacco cessation with AN/AI populations.

**Methods:**

The present “QUIT” protocol is a two-phase study that will occur at Southcentral Foundation (SCF), an Alaska Native-owned health system, serving 70,000 AN/AI people, based in Anchorage, Alaska. In Phase one, qualitative interviews with customer-owners (patients), providers and administrators (n = 36) and a 10-participant beta-test will be used to refine a strategy to implement NMR testing in the health system. Phase two will involve a single-arm pilot trial (n = 50) and qualitative interviews throughout data collection (n = 48) to evaluate the implementation strategy and explore the real-world acceptability and feasibility of NMR testing to guide tobacco cessation with AN/AI populations.

**Discussion:**

This study utilizes a community-based participatory approach to refine and implement a nicotine metabolism-informed smoking cessation program in a Tribal healthcare setting. The process and findings from this study will reflect the importance of customer-owner choice and honor the lived experience involved in quitting commercial tobacco. Pilot study data will inform the effect and sample sizes required for a future pragmatic trial of NMR-guided smoking cessation.

## Introduction

In Alaska, two-thirds of people who smoke commercial tobacco want to quit, and more than half of these individuals have tried to quit in the last year.^1^ Although there have been considerable advances in healthcare for American Indian and Alaska Native (AI/AN) peoples in recent decades,^2^ improving smoking cessation treatment remains an important priority in addressing health disparities. The prevalence of smoking among adults in the U.S. dropped from 95% in 1980 to 14% in 2020. Among the AI/AN population, however, rates remain among the highest in the nation at 23%. Furthermore, tobacco-related mortality is 60% higher among AI/AN people than white people in the U.S..^3^ Although the estimated number of people with tobacco-related health problems has gradually declined, there continues to be 60% higher smoking-attributable mortality (e.g., lung cancer, COPD, heart disease, and stroke) among AI/AN persons than among white people^3^, and quit rates are lower for AI/AN adults than others.^1^

Nicotine replacement therapy (NRT) remains the most frequently used first-line pharmacologic treatment for tobacco cessation.^4^ While several medications are currently available for tobacco cessation, including NRT, varenicline, and bupropion, selecting the most effective medication for an individual can be a lengthy process and include serious side effects. Nicotine metabolism can be affected by many things including environmental factors and genetic variations, specifically in the CYP2A6 allele.^5^ The nicotine metabolite ratio (NMR) is a genetic test that reveals how a person metabolizes nicotine and holds promise for identifying the most effective tobacco cessation medication with the fewest side effects.^6, 7^ The NMR biomarker identifies how “slow” or “fast” a person clears nicotine. For example, individuals with slow nicotine metabolism have been found to respond well to treatment with NRT, but individuals with normal or “fast” nicotine metabolism are significantly more likely to be successful in quitting tobacco using varenicline.^7^

Wells et al.^8^ demonstrated the feasibility of a metabolism-informed care process using ad hoc NMR testing to increase NMR-treatment match rates. Participants were informed by a nurse tobacco specialist of optimal smoking cessation treatments and encouraged to follow NMR-informed recommendations. They found that participants who were randomized to the metabolism-informed care group had higher medication matching (84%) compared to the usual-care group (58%).

A recent observational study of AI/AN adults starting the Quit Tobacco Program (QTP) at Southcentral Foundation found that 28% of participants demonstrated slow nicotine metabolism (NMR < 0.31), 66% had normal nicotine metabolism (NMR ≥ 0.31), and 6% had an NMR that was unquantifiable (either cotinine or 3-hydroxycontinine values were < 1.5 ng/mL at baseline).^9^ Of the total sample, 24% were smoking-abstinent at six weeks. A combined group of people with slow metabolism who used NRT and people with normal metabolism who used varenicline had a quit rate of 36.5%, a 13% higher quit rate than in the total sample. However in retrospective analyses, only 37% of participants chose a treatment that matched NMR-informed recommendations because no process was in place to inform them of the optimal treatment.^10^

The present study protocol aims to: 1) adapt a protocol to implement NMR testing for pharmacologic tobacco cessation treatment by conducting qualitative interviews with potential end users and stakeholders followed by a beta-test; and 2) conduct a single-arm pilot trial to assess the acceptability and feasibility of the intervention including evaluation of factors which may facilitate or impede the implementation of the intervention within the Alaska Tribal Health System (ATHS). We expect that establishing a culturally respectful testing process with customer-owners, and a logistically feasible clinical workflow for providers and leaders will support the implementation of an effective smoking-cessation intervention among AI/AN peoples in a real-world setting.

## Methods

### Study Overview

This study incorporates a Community-Based Participatory Research (CBPR) approach with AI/AN customer-owners, providers, and leaders to assess the acceptability and feasibility of a planned tobacco-cessation intervention.^8^ CBPR is a collaborative research approach that equitably involves community members, researchers, and other stakeholders in the research process and recognizes the unique strengths that each bring.

**Figure 1:**
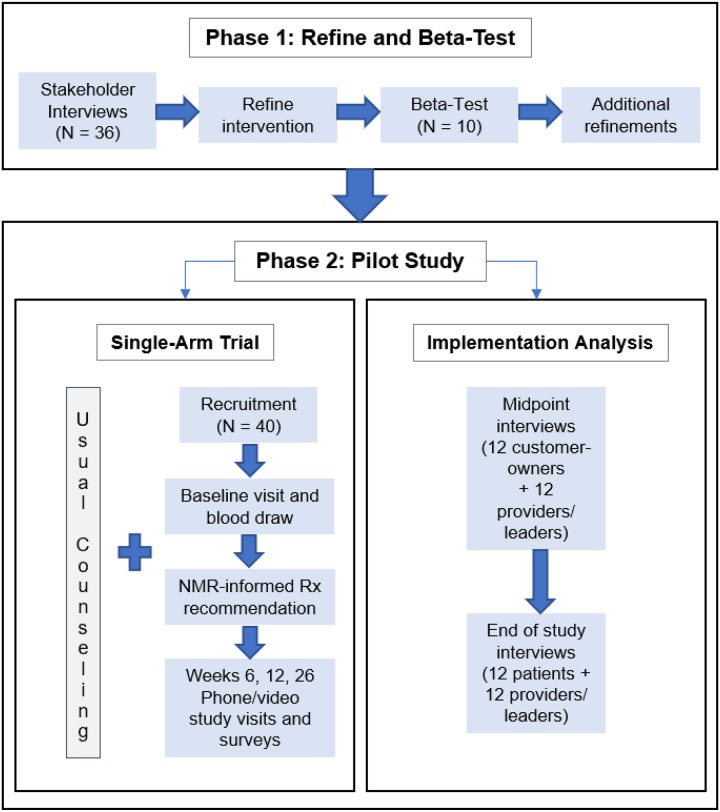
Study Overview

This mixed-methods study will be conducted in two phases (Fig. 1). Phase 1 is a qualitative study using individual, semi-structured interviews to elicit feedback about the acceptability and feasibility of using NMR to inform selection of pharmacologic treatment followed by a beta-test of the intervention. After refining study procedures in the beta-test, Phase 2 will pilot a single-arm trial and implementation analysis to examine factors which may impede or support implementation of the intervention within the Alaska Native Tribal health system. Our multidisciplinary team includes researchers, including AN/AI community members, with expertise in tobacco-cessation pharmacotherapy, public health, psychology, anthropology, intervention adaptation, community-engagement, and implementation science. We have also involved and appreciated AI/AN persons’ input and participation, including SCF customer-owners (i.e. SCF patients), SCF providers, and tribal health system leaders, in every phase of project development.

### Setting

SCF serves 70,000 AI/AN peoples in the Anchorage and Matanuska-Susitna Boroughs and 55 rural villages. Most study activities will take place in SCF-owned buildings on the Alaska Native Health Campus in Anchorage, Alaska (pop. ~300,000). Customer-owners interested in smoking cessation typically discuss this with their primary care providers before being referred to the Quit Tobacco Program (QTP). SCF has a dedicated department within Health Education called the Quit Tobacco Program (QTP) in which customer-owners can access cessation medications, behavioral counseling, or social support to help stop smoking.

### Regulatory Approvals

Our study was approved by the Alaska Area Institutional Review Board and SCF Research Review Committee.

### Participants

Customer-owner participants will complete some data collection activities at home for convenience. For Phase 1, semi-structured interviews will be conducted with eligible customer owners: AI/AN persons ≥ 18 years of age who are receiving services at SCF and use cigarettes as the main form of tobacco, smoked ≥ 1 cigarette per day over the last 30 days, are willing to make a quit attempt within 30 days of enrollment in the QTP, and have phone service. Six SCF clinical/administrative leaders and six providers will also be interviewed to understand practice needs and inform intervention materials and procedures. Clinic administrators and SCF leaders known to research staff will be emailed recruitment invitations, and the list of leaders will be reviewed by the Research Oversight Committee to ensure balanced workgroup representation.

Eligibility for Phase 2 beta-test customer owner participants will be the same as the Phase 1 interviews, plus having broadband internet connection on a mobile phone at home, work, or other location to complete smoking abstinence measures via phone or video teleconference. Interested individuals will be excluded from the beta-test if they participated in Phase 1 interviews, have participated in a smoking cessation program in the last three months, are contraindicated for the use of varenicline, have bleeding disorders, or are currently receiving cancer treatment. Care team staff eligibility for the beta-test includes being a healthcare professional at SCF and being directly involved in the care of a beta-test participant.

Eligibility for customer-owner participants in the Phase 2 pilot trial matches the beta-test; anyone who participated in an earlier phase of the study will be excluded. Phase 2 implementation interviews will be conducted with pilot trial participants, as well as SCF administrative leaders and clinical providers who have a customer-owner enrolled in the pilot trial.

### Phase 1 Interviews

Semi-structured interviews will be conducted with AI/AN customer-owners and SCF leaders and providers to refine the intervention materials and protocol. Using a sex-stratified purposive sample, we plan to interview approximately 36 participants to reach data saturation in the following groups: 24 customer-owner interviews (12 men, 12 women), six interviews with SCF leaders (e.g., administrators, clinical directors, tribal leaders), and six interviews with providers (e.g., primary care providers, pharmacists, tobacco treatment specialists, nurse case managers).

Separate interview guides will be developed for customer-owners and leaders/providers following the implementation frameworks of Reach, Effectiveness, Adoption, Implementation, Maintenance (RE-AIM) and Consolidated Framework of Implementation Research (CFIR) (Table 1).^11, 12^ Topics will include: (1) assessment of the needs and preferences regarding selection of tobacco-cessation medication; (2) perceived acceptability of the intervention and protocol, and procedures (communication of NMR result, timing of follow-up visits, remote vs in-person visits); (3) roles of treatment and study staff; (4) documentation process for NMR status and medication recommendation; and (5) recommendations for process and outcome measures. Interviews are expected to last up to one hour, and customer-owner interview participants will receive a $50 gift card for their time. SCF leaders and providers are paid employees and will not be compensated additionally by the study as interviews will take place during working hours. Qualitative analysis of the interview data will be used to refine the NMR-informed intervention prior to the beta-test. Qualitative data will be analyzed using ATLAS.ti.22 prior to descriptive thematic analysis.

### Phase 1 Beta-Test of NMR Implementation Strategy

A sex-stratified, purposive sample of 10 customer-owners will be enrolled in a beta-test of the NMR implementation strategy. The goal of the beta-test will be to refine the implementation strategy before the Phase 2 pilot. Phase 1 beta participants will be recruited through SCF primary care clinics and Quit Tobacco Program (QTP) referrals, word of mouth, and study flyers. Screening will occur by phone or in-person. Confirmation of eligibility and written informed consent will occur in-person or remotely online.

Following enrollment, participants will complete a demographic survey and have a blood sample (< 5ml) drawn prior to an intake with the QTP. Samples will be express shipped to a certified laboratory (Quest Diagnostics lab; Chantilly, VA) for NMR quantification, and results will be entered into the electronic medical record (EMR) and calculated as either slow (< 0.31) or normal (≥ 0.31) nicotine metabolism. Results are expected within 3–6 days of sample arrival at the laboratory. The appropriate therapeutic recommendation will be viewable by the care team in the EMR. Individuals with slow NMR will be recommended NRT patch. Individuals with normal metabolism will be recommended varenicline or bupropion, either alone or in combination with NRT gum or lozenge.

Although participants will be shown a list of their recommended medications, they will be able to select any of the provider-approved medications for which they are medically eligible. If an individual wishes to start medication immediately before NMR results are available, they will be recommended varenicline or bupropion (the recommendation for normal metabolizers) as NMR normal metabolizers are more common than slow metabolizers. Twelve weeks of medication will be dispensed by pharmacists through the standard clinical procedures, and participants will also have access to the usual non-pharmacologic treatments (e.g., behavioral counseling and support by phone) through the QTP. Six weeks after the participants’ quit date (set by the participant with the QTP), they will complete a follow-up survey and at-home smoking cessation tests to confirm biochemical abstinence. At-home smoking tests will be mailed to participants and will include an Ico Smokerlyzer^®^ (Bedfont Scientific) which will connect wirelessly to their smartphone and a NicAlert^™^ saliva test strip which is a small and inexpensive lateral flow immunoassay test strip that is activated by eight drops of saliva. Also included in the mailed at-home test kits are step-by-step lay language instructions re-written from the original instructions. The test will also include photos of study staff self-administering both tests. Beta-test participants will receive a $50 gift card for the baseline blood draw and $75 for the follow-up study visit.

After customer-owner participants have completed the clinical process of NMR-informed medication recommendation, semi-structured interviews will be conducted with up to 20 care team members (two for each customer-owner participant). Recruitment emails will be sent to staff known to have been involved in the care of beta-test participants, and verbal consent will be obtained prior to the interview. Interviews will not be recorded, but detailed notes will be taken by the interviewer. To accommodate staff schedules, staff participants can complete a survey in place of an interview. Best-test data including demographic, acceptability questions, Electronic Health Record data and follow-up measure results will be analyzed using descriptive statistics. Content analysis will be used to analyze the interview data, brief study notes from the CO beta-test study visits and individual and group staff interviews.

### Phase 2 Pilot Trial and Implementation Evaluation

Following Phase 1, measures may be modified to reflect the results of the beta test, and a single-arm pilot trial will be conducted with 50 customer-owners to evaluate the refined implementation strategy and explore the effectiveness of the NMR for optimizing individual tobacco cessation treatment. The sample size was determined assuming a 60% retention rate to ensure at least 30 participants have follow-up data at 26 weeks.^13–15^ Follow-up visits will be conducted as follows: at 6 weeks after starting pharmacologic treatment (to assess intervention acceptability, nicotine dependence/withdrawal, side effects, and medication adherence); at 12 weeks, the planned end of pharmacologic treatment (to assess all 6-week measures plus acceptance of study processes and biochemically verified quit status); and at 26 weeks (to assess biochemically verified quit status). Pilot-trial participants will receive the same compensation as beta-test participants for completing study procedures.

Phase 2 will also explore barriers and facilitators to implementation of the intervention within SCF to prepare for a future pragmatic trial. At the mid-point and end of the pilot trial (13 and 26 weeks), we will conduct brief, semi-structured interviews with six SCF leaders (e.g., administrators, clinical directors, tribal leaders) and six randomly selected providers who had a customer-owner who participated in the pilot trial. Interviews will assess the process of using metabolism-informed care and challenges in adhering to the treatment recommendations. Brief, semi-structured interviews will also be conducted with 12 trial participants at the mid-point and end of the pilot trial, randomly selected within sex strata, about the intervention and treatment fidelity. Trial participants will be paid $25 per interview for a total of up to $50, but SCF staff will not receive additional compensation because interviews will be conducted during paid working hours.

### Outcomes and Statistical Methods

Phase 1 qualitative interviews will be thematically analyzed to inform the development of a strategy to implement NMR in tobacco cessation treatment. Quantitative and qualitative data collected in the beta-test will be used to refine study procedures in the pilot trial. The primary outcomes of the pilot trial are acceptability (Table 2) and feasibility (Table 3) of the intervention and its procedures, as assessed by study process metrics and completeness of clinical measures. Descriptive statistics will be calculated to describe sample demographics, clinical factors, tobacco history, and other co-variates. Given the nature and sample size of the single-arm pilot trial, all analyses will be treated as exploratory.

Acceptability and satisfaction will be assessed by calculating the mean and standard deviation of Likert-scale survey responses of items such as: timeliness and clarity of the NMR recommendation, medicine dispensation timeliness, ease of understanding trial procedures, and overall satisfaction with the intervention, compensation, and study participation. Feasibility and completeness of trial measures will be calculated based on the proportion of eligible screened participants who enroll, accrual and retention rates, proportion of enrolled participants who complete follow-up visits, proportion who use the NMR-informed pharmacologic treatment as recommended, and data completeness. Smoking abstinence at follow-up visits will also be compared to assess intervention quality and robustness. Data from this pilot trial will be used to estimate possible effect size and calculate sample size required for a future pragmatic trial.

## Discussion

NMR-informed medication selection for tobacco cessation is supported by a growing body of evidence.^6–8^ Over four years, our study team worked with Tribal health system stakeholders to design this project, and input was provided by AI/AN advisors to ensure that using NMR testing in an Alaska Tribal healthcare center would be feasible, sustainable, culturally appropriate, and useful for both the organization and recipients of tobacco cessation treatment. While pharmacologic treatment for smoking cessation should be tailored to an individual’s rate of nicotine metabolism for the best results and fewest side effects, there is currently no clinical workflow to provide optimized treatment in the Alaska Native healthcare system. In addition, as the NMR is a phenotype (i.e., a biomarker) of genetic variation,^5, 16^ the acceptability of metabolic testing must also be assessed given concerns about genetic research among AI/AN peoples.^17^ Thus, the primary endpoint of this study is not the efficacy of NMR-informed pharmacologic treatment on cessation, but rather the acceptability and feasibility of implementing NMR-informed pharmacologic treatment into the clinical workflow in an Alaska Native healthcare system.

To ensure that the implementation of NMR-guided tobacco cessation treatment is acceptable to customer-owners, providers, and administrators, we will use a mixed methods approach following the RE-AIM framework. Quantitative data collected in the Phase 2 pilot trial and qualitative interviews will provide insight into how NMR-guided tobacco cessation treatment can be engaged in a way that is feasible and scalable in other AI/AN healthcare systems.

Our study had several strengths, most importantly the use of a CBPR mixed-method approach to ensure the project is done in an equitable and collaborative way and that we incorporate qualitative in-depth feedback from AI/AN peoples. The refined intervention will thus honor the specific lived experience and culture of Customer-Owners who smoke and have tried to quit by engaging health system stakeholders and participants who currently use commercial tobacco and providers who work with those wishing to quit. While NMR-informed cessation treatment selection and its implementation show promising results in other populations, this is the first study to pilot its use with AI/AN peoples. Prior studies utilized trial designs in which participants were assigned to a treatment arm based on NMR results to control confounding variables. However, given the cultural importance of agency and choice, participants in this pilot trial will be allowed to choose their preferred treatment, even if different from the NMR-informed recommendation. Our primary focus on feasibility, proportion of participants who use the recommended treatment, and rate of adherence to medication usage represents a key strength in evaluating the implementation of NMR testing in this population. Completeness of trial measures and data analysis are expected to thoroughly inform future trial design.

The study inclusion criteria require participants to have access to broadband internet which could be a barrier for some populations, especially given that smoking rates are exceptionally high among people who are unhoused.^18^ Additionally, as this study is a single-arm pilot to assess acceptability and feasibility, we will treat all analyses as exploratory. While there will be no formal analysis of pharmacologic efficacy, prior research has already established the efficacy of NMR testing in informing pharmacologic treatment for smoking cessation. The planned pilot trial is expected to inform the design of a future pragmatic trial and has the potential to support tobacco cessation work among other AI/AN communities.

## Figures and Tables

**Table 1:** Application of the RE-AIM Framework to Implement Metabolism-Informed Pharmacologic Smoking Cessation Treatment

Domain	Existing Knowledge Gaps	Quantitative Data from Pilot	Qualitative Data from Interviews Across Aims
**REACH**: Proportion willing to participate in the intervention	Uptake on institutional level unknown Extent customer-owners and providers willing to participate in QUIT intervention unknown	% customer-owners, providers participating (data from Aim 2)	Factors influencing acceptability of intervention to SCF leaders, customer-owners, and providers (data from Aim 1)
**EFFECTIVENESS**: Ability to improve outcomes	Prior trials confirmed effectiveness in other smoking populations, but not diverse tobacco users from an AI/AN community sample. “Real world” effectiveness still to be determined Definitive answers await the full-scale pragmatic trial	Provider parameters for estimate of sample size for pragmatic trial assessing effectiveness.	NA
**ADOPTION**: Proportion who actually use the intervention	High-level interest from health system leaders, but adoption by frontline providers unknown Will customer-owners select the recommended pharmacologic treatment?	Variation in match between NMR and pharmacologic treatment across providers and clinics (data from Aim 2)	Barriers and facilitators to “real world” adoption and variability across provider and clinics (data from Aim 3)
**IMPLEMENTATION**: Fidelity and consistency of use	Consistency of use requires timely lab draw in primary care. Will use vary by provider/clinic?	Ongoing fidelity checks during Aim 2	Providers’ and customer-owners’ challenges with maintaining fidelity (data from Aim 3)
**MAINTENANCE**: Consistency over time and settings	Maintenance of the intervention is unknown Because some of the same providers will participate in the full-scale pragmatic trial, we will assess maintenance later, in that trial	NA	NA

**Table 2:** Pilot Study Acceptability Measures

Acceptability Measures	Data Source
1) timeliness of recommendation	Survey*, EHR
2) composition of clinical team for tailoring treatment	Survey
3) clarity of recommendation	Survey
4) timeliness of medication dispensation	Survey, EHR
5) overall assessment of metabolism-informed care processes	Survey
6) ease of understanding baseline and follow-up trial measures	Survey
7) willingness to recommend participation to a family or friend	Survey
8) satisfaction with the intervention	Survey
9) satisfaction with study processes	Survey
10) satisfaction with data collection methods	Survey
11) satisfaction with compensation	Survey
12) satisfaction with study participation	Survey

* Surveys will be completed by customer-owners.

**Table 3:** Pilot Study Feasibility Measures

Feasibility Measures	Data Source
1) # participants screened, per month	Study Records
2) # participants consented, per month	Study Records
3) # participants enrolled, per month	Study Records
4) # with quantifiable NMR lab results in EHR	EHR
5) # given recommendation by pharmacist	Survey, EHR
6) % recommendation matches CDS	EHR
7) # dispensed treatment	EHR
8) # dispensed recommended treatment	EHR
9) rates of adherence to prescribed treatment	Survey
10) % retained in study	Study Records
11) % of planned study visits completed	Study Records
12) duration of study visits	Study Records
13) % that needed to be rescheduled	Study Records
14) % that initiated contact with study team during follow-up period	Study Records

## Data Availability

The data that support the findings of this study are available from the corresponding author, upon reasonable request and approval of the applicable Tribal research review committees.
